# Plasma proANP and SDMA and microRNAs are associated with chronic mitral regurgitation in a pig model

**DOI:** 10.1530/EC-13-0051

**Published:** 2013-09-24

**Authors:** Susanna Cirera, Sophia G Moesgaard, Nora E Zois, Nathja Ravn, Jens P Goetze, Signe E Cremer, Tom Teerlink, Páll S Leifsson, Jesper L Honge, J Michael Hasenkam, Lisbeth H Olsen

**Affiliations:** Department of Veterinary Clinical and Animal Sciences, Faculty of Health and Medical SciencesUniversity of CopenhagenFrederiksberg CDenmark; 1 Novo Nordisk A/SMaaloevDenmark; 2 Department of Clinical BiochemistryCopenhagen University Hospital RigshospitaletCopenhagenDenmark; 3 Department of Cardiothoracic and Vascular SurgeryAarhus University HospitalAarhusDenmark; 4 Department of Clinical BiochemistryAarhus University HospitalAarhusDenmark; 5 Department of Veterinary Disease Biology, Faculty of Health and Medical SciencesUniversity of CopenhagenFrederiksberg CDenmark; 6 Department of Clinical ChemistryVU University Medical CenterAmsterdamThe Netherlands

**Keywords:** pig, mitral regurgitation, proANP, SDMA, microRNA

## Abstract

**Objective:**

Non-ischemic mitral regurgitation (MR) is primarily caused by myxomatous mitral valve (MV) disease leading to adaptive remodeling, enlargement, and dysfunction of the left ventricle. The aim of this study was to examine the regulation of plasma markers and several cardiac key genes in a novel porcine model of non-ischemic MR.

**Methods and results:**

Twenty-eight production pigs (*Sus scrofa*) were randomized to experimental MR or sham surgery controls. MR was induced by external suture(s) through the posterior MV leaflet and quantified using echocardiography. The experimental group was subdivided into mild MR (mMR, MR=20–50%, *n*=10) and moderate/severe MR (sMR, MR >50%, *n*=6) and compared with controls (CON, MR ≤10%, *n*=12). Eight weeks postoperatively, follow-up examinations were performed followed by killing. Circulating concentrations of pro-atrial natriuretic peptide (proANP), l-arginine, asymmetric dimethylarginine, and symmetric dimethylarginine (SDMA) were measured. MV, anterior papillary muscle, and left ventricular free wall tissues were collected to quantify mRNA expression of *eNOS* (*NOS3*), *iNOS* (*NOS2*), *MMP9*, *MMP14*, *ANP* (*NPPA*), *BNP* (*NPPB*), and *TGFB1*, *2*, and *3* and five microRNAs by quantitative real-time PCR. Pigs with sMR displayed markedly increased plasma proANP and SDMA concentrations compared with both controls and mMR (*P*<0.05). The expression of all genes examined differed significantly between the three localizations in the heart. *miR-21* and *miR-133a* were differently expressed among the experimental groups (*P*<0.05).

**Conclusions:**

Plasma proANP and SDMA levels and tissue expression of *miR-21* and *miR-133a* are associated with severity of chronic MR in an experimental porcine model.

## Introduction

Non-ischemic mitral regurgitation (MR) is defined as incomplete coaptation of the mitral valve (MV) resulting in backflow of blood from the left ventricle to the left atrium during systole. MR is primarily caused by myxomatous MV disease (MMVD), leading to adaptive remodeling, enlargement, and subsequent dysfunction of the left ventricle (1). Severe MR may need surgery, and if left untreated, it can cause heart failure or serious arrhythmia. In the USA, about 18 000 patients undergo MV surgery every year (2). Still, the pathophysiology of MMVD is incompletely understood and further elucidation of the mechanisms involved herein is needed to improve diagnostics and therapy. It is unclear what initiates the degenerative cellular changes in the MV and myocardium leading to the disease and the extent to which hemodynamic features of MR affect cellular factors.

Animal models of naturally occurring and experimentally induced MR are described. MMVD is the most common cause of MR in dogs and the pathology is similar to primary MV prolapse in humans (3). Narrowing of intramural vessels in the left ventricle correlates with echocardiographic measurements of MR severity in dogs, indicating that cellular processes in the myocardium might also have an impact on MMVD progression and vascular function (4).

Several diagnostic techniques have been developed to investigate the etiology and pathophysiology of MR including circulating biomarkers. Plasma concentrations of atrial and brain natriuretic peptides (ANP and BNP) are useful in the diagnosis of canine heart failure and may also aid in the detection of severe preclinical MR (5). Asymmetric dimethylarginine (ADMA) is an endogenous inhibitor of nitric oxide synthase (NOS) and several studies in human patients show correlation between changes in plasma ADMA concentrations, endothelial dysfunction, and cardiovascular risk (6).

Several studies have highlighted the role of cellular markers including matrix metalloproteinases (MMPs), transforming growth factor β (TGFβ) isoforms, NOS, and natriuretic peptides (7, 8, 9, 10). Moreover, microRNAs have emerged as new players in many developmental and pathological processes, i.e. cardiac development and heart hypertrophy.

The disadvantage of the clinical studies in patients and animals with naturally occurring MR is the difficulty in controlling factors such as age, lifestyle, treatments, and other concurrent diseases. However, these factors can be avoided in studies of experimental animal models. By using large animal models (i.e. pigs), physiologically and genetically closer to humans than rodents, improved understanding of disease and potential testing of new therapeutic and diagnostic approaches ensues (11, 12).

In this study, a novel porcine model of non-ischemic chronic MR provides a platform for further investigations of the hemodynamic features of chronic MR and the association with plasma markers and cellular processes in the heart. We have characterized the model by examining pro-atrial natriuretic peptide (proANP), l-arginine, ADMA, and symmetric dimethylarginine (SDMA) concentrations in plasma and the regulation of several valvular and myocardial key genes (*NOS3* (*eNOS*), *NOS2* (*iNOS*), *MMP9*, *MMP14*, *TGFB1*, *TGFB2*, *TGFB3*, *NPPA* (*ANP*), and *NPPB* (*BNP*)) and key microRNAs (*miR-1*, *miR-21*, *miR-23a*, *miR-133a*, and *miR-195*).

## Materials and methods

### Experimental animals

Forty-three female Danish Landrace/Yorkshire pigs (*Sus scrofa*) weighing ∼20 kg were randomized to either intervention (MR, *n*=30) or control (CON, *n*=13) groups. The intervention group had surgically introduced MR, while the control group underwent sham operation.

Animal handling was performed according to the guidelines from the Danish Inspectorate for Animal Experimentation and adhered to the principles provided in the Guide for Care and Use of Laboratory Animals and the Directive 2010/63/EU of the European Parliament. If the animals suffered from refractory pain or failed to thrive, they were killed.

### Anesthesia

Animals were premedicated with i.m. midazolam 0.5 mg/kg and ketamine 5 mg/kg. Anesthesia was initiated by i.v. administration of hypnomidate 0.5 mg/kg. Animals were intubated endotracheally and coupled to a ventilator. Cefuroxime 750 mg was i.v. administered both pre- and postoperatively to prevent infection. General anesthesia was maintained with inhalational sevoflurane (3%) and analgesia was induced by bolus administration of fentanyl 8 μg/kg. Muscle paralysis was achieved by administrating esmeron 2.5 mg/kg. Adequacy of anesthesia was monitored by continuous ECG, pulse oximetry, and capnometry. Postoperative analgesia was achieved by flunixine 0.4 mg/kg once daily for 3 days. Buprenorphine 0.6 mg was administered i.m. if deemed necessary.

### Surgical technique

Surgical induction of MR is described in detail elsewhere (13). Briefly, from a left-sided thoracotomy, the posterior mitral leaflet was fixated by one to three sutures applied through the myocardium of the left ventricle, ∼1 cm dextrolaterally and 1 cm apically of the coronary sinus, and advanced through the P2 segment of the posterior leaflet and the left atrium. Before closure, presence of MR was assessed echocardiographically and by digital thrill palpation. The control group underwent sham operation with left thoracotomy and opening of the pericardium. The posterior MV leaflet was left untouched in this group, and after echocardiographic assessment to verify MV competence, the chest was closed in layers. When hemodynamic stability was achieved and the pigs were breathing spontaneously, they were transported to the farming facilities.

### Echocardiography

Standardized images were acquired in accordance with the recommendations for transthoracic echocardiography in left and right lateral recumbency using a Vivid E9 echocardiograph equipped with a 2.0–5.0 MHz transducer (GE Healthcare, Horten, Norway). Echocardiograms were performed preoperatively and at follow-up using the same protocol as described elsewhere (13). Data analysis was performed by one investigator using Echopac (GE Healthcare). All values reported were averages of three consecutive heart cycles. Using 2D-guided M-mode performed on the short axis view at the level of the chordae tendineae, left ventricular diastolic internal dimensions (LVIDd) were measured. MR was quantified (to the nearest 5%) as the maximum area of the regurgitant jet measured in percentage of the area of the left atrium in two orthogonal planes (parasternal and apical four-chamber views).

### Reexamination and tissue sampling

After a follow-up period of 8 weeks, each animal was anesthetized and reexamined using the same anesthetic and echocardiographic protocol as at baseline. Next, a sternotomy was performed and blood samples were taken by venipuncture of the pulmonary artery and centrifuged within 30 min of collection. Plasma samples were stored at −80 °C until analysis. Pigs were killed by exsanguination, after which the heart was eviscerated and weighed. Immediately thereafter, the heart was dissected and samples from the posterior MV leaflet, anterior papillary (AP) muscle, and left ventricular (LV) free wall were stored in RNAlater for 24 h at 4 °C followed by storage at −20 °C until RNA extraction. Samples from the anterior MV leaflet were fixed in formalin and embedded in paraffin for histopathological examination following standard procedures.

### Plasma analyses

Plasma concentrations of endogenous proANP were measured using an immunoassay developed recently (14, 15). The inter-assay coefficient of variation (CV) was always <15%.

Plasma concentrations of l-arginine, ADMA, and SDMA were determined simultaneously by HPLC as described previously (16), using modified chromatographic separation conditions (17). The l-arginine, ADMA, and SDMA had an analytical recovery of 98–102% and the inter-assay CV was <3% for all compounds. Routine hematology and biochemical analyses were also performed.

### RNA extraction and quality assessment

Approximately 50 mg tissue stored in RNAlater was used for total RNA extraction using the RNeasy fibrous tissue mini kit (Qiagen Gmbh, Hilden, Germany). The RNA was quantified using a NanoDrop 1000 machine (Thermo Fisher Scientific Inc, Waltham, MA, USA). The RNA integrity was evaluated on an Experion system (Bio-Rad, Hercules, CA, USA) using Experion l RNA StdSens Analysis Kit (Bio-Rad). All samples showed excellent RNA quality indexes (RQIs) with values over 6.8 (only one sample, SG17 valve had a RQI=4.7). In detail, the average RQI value for the MV samples was 8.7±1.064, for the LV samples 9.0±0.68, and for the AP samples 9.0±0.35.

### cDNA synthesis

Two cDNA replicates were made for each RNA sample. Shortly, 1 μg total RNA was reverse transcribed at 42 °C using Improm-II reverse transcriptase (Promega, Madison, WI, USA) and a mixture 3:1 of random hexamers: OligodT. For microRNA detection, the cDNA synthesis was performed as described in reference (18). All cDNA samples were diluted 1:8 in dH_2_O prior to use in quantitative real-time PCR (qPCR).

### qPCR primers

Primer sets for *MMP1*, *MMP9*, *MMP14*, *ANP*, *BNP*, *TGFB1*, *TGFB2*, and *TGFB3* were designed using Primer 3 Software (http://bioinfo.ut.ee/primer3-0.4.0/). Primers were designed over introns if possible. Primers for the *eNOS* and *iNOS* genes were taken from reference (19). Primers for the *HPRT* (*HPRT1*), *TBP*, and *RPL4* (reference genes) were taken from reference (20).

Primers for the microRNAs (*let7a*, *miR-16*, *miR-21*, *miR-23a*, *miR-1*, *miR-133a*, and *miR-195*) were designed as described by reference (18) (primer sequences, amplicon size, and PCR efficiencies are listed in Supplementary Table 1, see section on supplementary data given at the end of this article).

### Quantitative real-time PCR

QuantiFast SYBR Green PCR Kit (Qiagen) was used for the qPCR amplification. Briefly, in a total volume of 10 μl, 2 μl dH_2_O, 5 μl 2× QuantiFast mastermix, 0.5–1 μl 10 μM forward primer, 0.5–1 μl 10 μM reverse primer, and 1μl 1:8 diluted cDNA were mixed in white PCR plates (Thermo Fisher Scientific). The qPCRs were performed on a Mx3000 machine (Stratagene, Santa Clara, CA, USA). The PCR thermal profile was as follows: 95 °C for 5 min, 40 two-step cycles of 95 °C for 10 s, 60 °C for 30 s, and a melting curve analysis (55–95 °C) was performed in the last cycle to evaluate specificity. Assay efficiency for each gene was calculated using a dilution serial of the purified PCR product generated by each specific primer pair. The baseline adjustment method of the Mx3000/MxPro (Stratagene) Software was used to determine the *C*q in each reaction. Expression data were analyzed using the GenEx Pro Software (Multid Analyses AB, Göteborg, Sweden): *C*q values were normalized to the geometric mean of the reference genes (*HPRT*, *RPL4*, and *TBP* for mRNAs and *let7a* and *miR-16* for microRNAs) and the average was calculated for each pair of cDNA replicates. Finally, relative quantities were calculated for each marker in relation to the lowest expressed sample.

### Statistical analysis

Statistical analysis was performed using SAS Statistical Software version 9.2 (SAS, Cary, NC, USA) and GraphPad Prism version 5 (GraphPad Prism, La Jolla, CA, USA). A one-way ANOVA with Bonferroni's multiple comparisons test or a Kruskal–Wallis test was used to test the difference in plasma markers between the groups. Linear regression analyses were performed to assess the correlation between plasma markers (proANP and SDMA) and echocardiographic markers (MR and LVIDD) and mRNA markers.

Linear mixed effects models were used to evaluate the influence of heart locations (MV, LV, and AP) and experimental groups (CON, mild MR (mMR), and moderate/severe MR (sMR)) on the expression levels for the different genes (nine protein-coding genes and five microRNAs). The variable ‘pig’ was included as random effect. The interaction between heart location and experimental group was included in the respective models. All models were tested for homogeneity and normality of the residuals by inspection of histograms, residual plots, and QQ plots. A *P* value <0.05 was considered significant. If significant overall effects of group and/or heart location were found, *post hoc* comparisons were performed using *t*-tests with Tukey corrections of the *P* values.

Principal component analysis (PCA), which reveals the internal structure of the data, was applied to autoscaled log_2_ expression values of all investigated genes and was performed in GenEx (Multid Analyses). Each gene was autoscaled to give all genes equal weight in the clustering algorithm.

## Results

### Model validation

The model has been described previously (13). Briefly, 35 pigs survived 8 weeks. Ten percent MR was chosen as the cutting point (as 10% was the upper limit for naturally occurring MR in the control group). Five pigs were excluded because of failure to induce MR above 10%. Two animals were excluded due to mediastinal infection with implication of the heart. Accordingly, 28 pigs were subjected to further analysis: 12 control pigs (CON) had MR ≤10%, ten intervention pigs had mMR (10%<MR≤50%), and six pigs had sMR (MR >50%). A significant increase in LVIDd was seen in the mMR group and the sMR group compared with CON, and LV weight differed significantly between the CON and the sMR groups (see (13), Table 1).

The histopathological findings are described in detail elsewhere (Cremer SE, Zois NE, Moesgaard SG, Ravn N, Cirera S, Honge JL, Smerup MH, Hasenkam JM, Sloth E, Leifsson PS *et al.*, 2013, unpublished observations). Briefly, of the 28 valvular samples, four could not be assessed histologically due to either insufficient material or lack of free margin. The severity and distribution of lesions differed between pig groups. Minor lesions consisted of small proliferations of endocardial and subendocardial cells and focal subendocardial fibrosis, and moderate and severe lesions consisted of foci of inflammatory cells and extensive proliferations of endothelial and subendothelial cells (Cremer SE, Zois NE, Moesgaard SG, Ravn N, Cirera S, Honge JL, Smerup MH, Hasenkam JM, Sloth E, Leifsson PS *et al.*, 2013, unpublished observations; Table 2 shows the severity and distribution of lesions among the different groups).

### Plasma analyses

**Figure 1:**
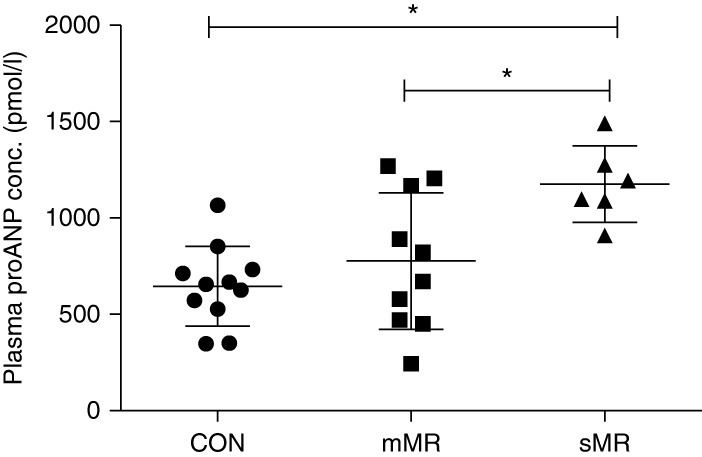
Plasma concentrations of pro-atrial natriuretic peptide (proANP) measured 8 weeks following surgically induced MR. CON, control; mMR, mild MR (10%<MR≤50%); sMR, moderate/severe MR (MR >50%); bars represent mean±s.d. *P<0.05.

**Figure 2:**
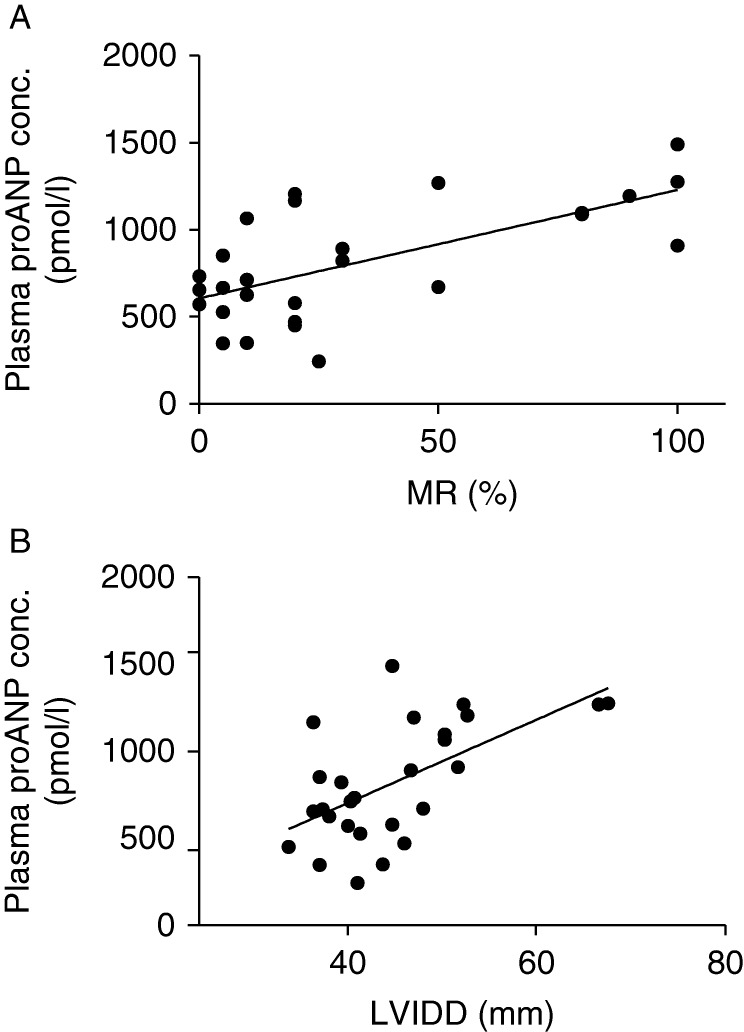
Increasing plasma concentrations of pro-atrial natriuretic peptide (proANP) correlated significantly with increasing MR (*P*=0.002, *R*^2^=0.42) and left ventricular end-diastolic diameter (LVIDD; *P*=0.001, *R*^2^=0.35) measured 8 weeks following surgically induced MR.

**Figure 3:**
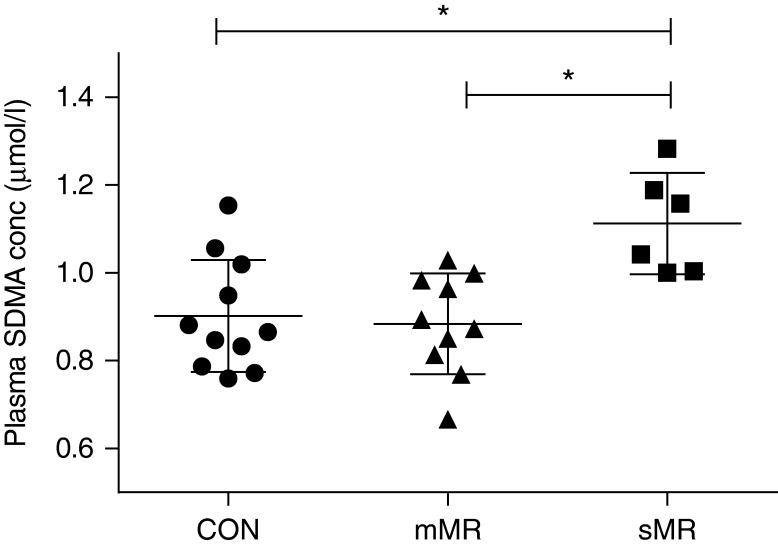
Plasma concentrations of symmetric dimethylarginine (SDMA) measured 8 weeks following surgically induced MR. CON, control; mMR, mild MR (10%<MR≤50%); sMR, moderate/severe MR (MR >50%); bars represent mean±s.d. *P<0.05.

**Figure 4:**
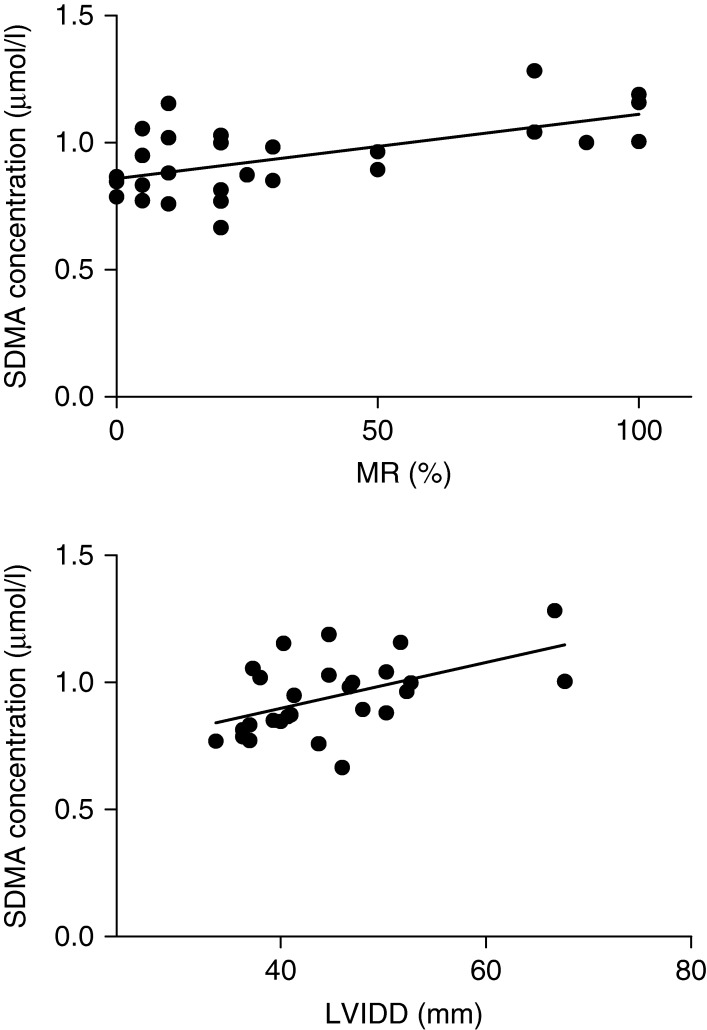
Increasing plasma concentrations of symmetric dimethylarginine (SDMA) correlated significantly with increasing MR (*P*=0.001, *R*^2^=0.35) and left ventricular end-diastolic diameter (LVIDD; *P*=0.006, *R*^2^=0.26) measured 8 weeks following surgically induced MR.

Plasma concentrations were analyzed in all pigs except for one control pig. There was a significant increase in plasma proANP concentration in pigs with sMR compared with mMR and control pigs (*P*=0.003, Table 1, Fig. 1). Furthermore, there was a significant correlation between increasing plasma proANP concentrations and increasing percentage MR (*P*=0.002, *R*^2^=0.42) and LVIDD (*P*=0.001, *R*^2^=0.35) (Fig. 2). Plasma SDMA concentration was significantly higher in pigs with sMR compared with mMR and control pigs (*P*=0.003, Table 1, Fig. 3), and a significant correlation between plasma SDMA concentration and increasing MR (*P*=0.001, *R*^2^=0.35) and LVIDD (*P*=0.006, *R*^2^=0.26) was found (Fig. 4). No difference among groups was found for plasma l-arginine and ADMA concentrations (Table 1). No decisive abnormalities or differences between groups were found in the hematology and biochemical analyses performed.

### Quantitative real-time PCR

**Figure 5:**
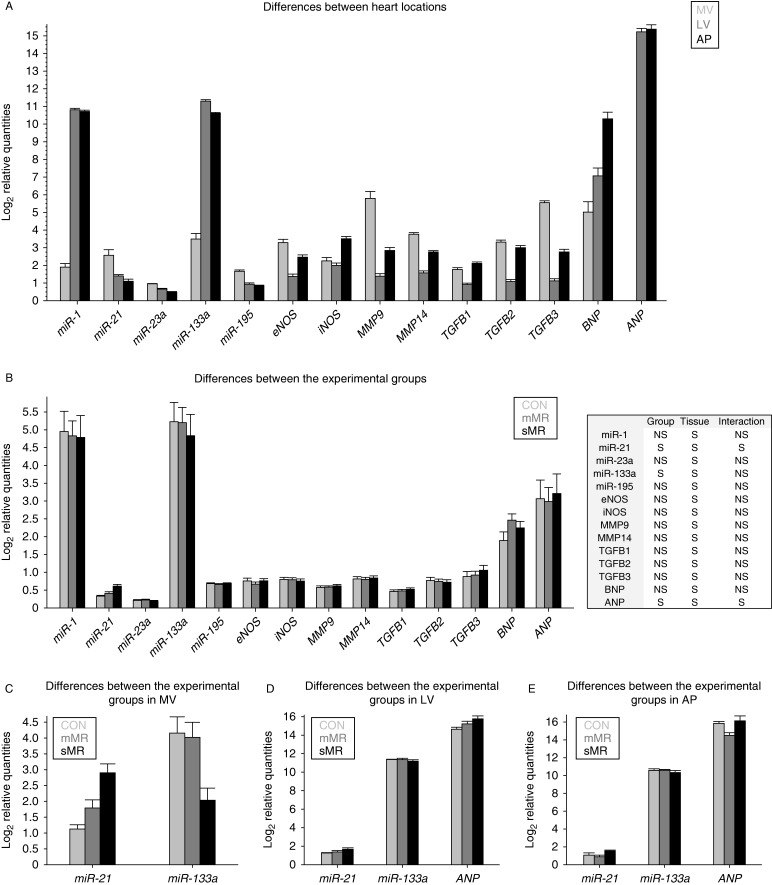
(A) Differences of expression between the heart locations (MV, LV, and AP) in all the genes in all the animals. (B) Differences of expression between the experimental groups (CON, mMR, and sMR) in all the genes in the three heart locations. (C) Differences of expression between the experimental groups (CON, mMR, and sMR) in *miR-21* and *miR-133a* genes in the MV location. The *ANP* gene was excluded from the analysis for the MV location (see text). (D) Differences of expression between the experimental groups (CON, mMR, and sMR) in *miR-21*, *miR-133a*, and *ANP* genes in the LV location. (E) Differences of expression between the experimental groups (CON, mMR, and sMR) in *miR-21*, *miR-133a*, and *ANP* genes in the AP location. Data used to construct these figures were log-transformed raw qPCR data and the bars represent the mean values of each experimental group or heart location for each specific gene. s.e.m. is depicted by error bars. Table next to (B). S, significant; NS, not significant.

Tissue from the three locations of the heart (MV, LV, and AP) from six control pigs (with the lowest MR, all ≤5%), ten mMR pigs, and six sMR pigs were used for qPCR analysis (see Fig. 5).

#### Protein-coding genes

qPCR results for the *MMP1* gene were under the limit of detection and were excluded from the analysis. The other nine genes showed significant differential expression between the three heart localizations (see Fig. 5A). Only one gene, *ANP*, showed a significant difference between experimental groups (see Fig. 5B).

##### ANP

There was a big variation between samples in the MV heart location. For this tissue, six samples belonging to the three experimental groups showed levels of expression under the limit of detection and results from the two cDNA replicates of these six samples were in complete agreement, pointing to real biological variation of the valvular expression levels of the *ANP* gene and not to a technical artifact. Therefore, we excluded data for the MV location for further statistical analysis. Subsequently, we compared the three experimental groups in the two remaining heart locations and found a significant interaction between heart location and experimental group (*P*=0.02). *Post hoc* analyses revealed significantly higher expression in the AP in the sMR group compared with the mMR group (*P*=0.03) but not when compared with the CON group.

#### MicroRNAs

All microRNAs showed a significant differential expression between the three heart locations (see Fig. 5A). For two of the microRNAs, *miR-21* and *miR-133a*, the interaction between experimental group and heart location was statistically significant, indicating that the microRNAs at the different heart locations were also influenced by experimental group (see Fig. 5B, C, D and E).

##### miR-21

A significant effect of the interaction between experimental group and heart location (*P*=0.02) was found. Within the sMR group, there was a significantly higher expression in the MV compared with the LV (*P*=0.002) and the AP (*P*=0.0003). Similarly, there was a significantly higher expression in the MV than in the AP (*P*=0.007) within the mMR group. The valvular expression in the sMR group was significantly higher than the valvular expression in the CON (*P*<0.0001) and the mMR groups (*P*=0.01). Moreover, the valvular expression in the sMR group was significantly higher than the expression in the LV of both the CON (*P*=0.0002) and the mMR groups (*P*=0.0002) and also higher than the expression in the AP of the CON (*P*<0.0001) and mMR groups (*P*<0.0001). The increase in valvular expression of miR-21 correlated significantly with the increase in plasma proANP (*P*=0.01, *R*^2^=0.30).

##### miR-133a

There was an effect of experimental group (*P*=0.0061) and location (*P*<0.0001) but not the interaction between them. MV had a significantly lower expression than LV (*P*=0<0.0001) and AP (*P*<0.0001). LV had a significantly higher expression than AP (*P*<0.0001). sMR had significantly lower expression than both CON (*P*=0.0149) and mMR (*P*=0.0103).

**Figure 6:**
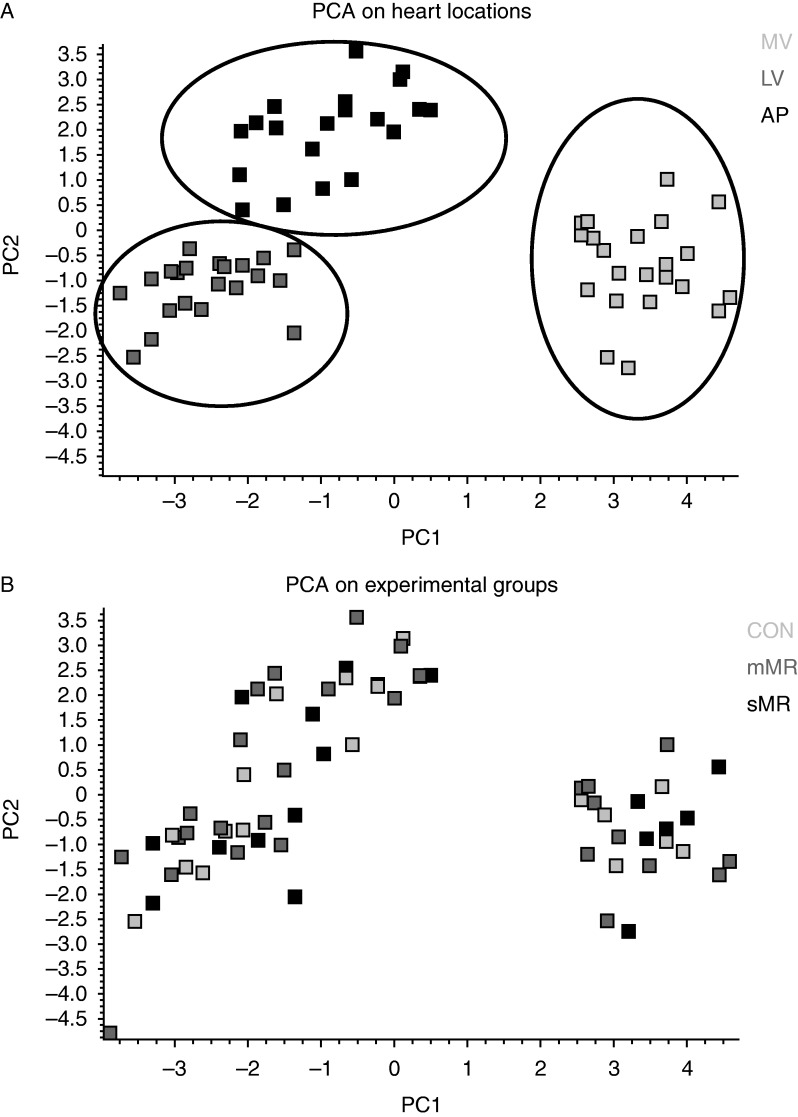
PCA of the qPCR data. A two-dimensional plot (PC1 and PC2) is shown. (A) PCA on heart locations and (B) PCA on experimental groups. Scaling of log_2_ relative quantities for all genes was included in the analysis.

The results of the PCA are shown as the two-dimensional contribution scores for component numbers 1 and 2 (PC1 and PC2; see Fig. 6). The PCA analysis of all 14 investigated genes was able to differentiate between the three heart locations, MV, LV, and AP (Fig. 6A) but not between the experimental groups (Fig. 6B).

## Discussion

This study evaluated biochemical regulation of plasma markers and key genes in a novel porcine model of chronic, non-ischemic MR induced by simple external immobilization and fixation of the posterior MV leaflet.

LV hypertrophy and enlargement was demonstrated in the sMR group compared with the CON group (13), and the hemodynamic changes also led to an increase in the severity of histopathological changes of the MV in the MR groups compared with the CON group (Cremer SE, Zois NE, Moesgaard SG, Ravn N, Cirera S, Honge JL, Smerup MH, Hasenkam JM, Sloth E, Leifsson PS *et al.*, 2013, unpublished observations). Plasma proANP concentrations were significantly increased in the sMR group compared with both mMR and controls. The increases in plasma proANP concentration correlated with increases in MR and LVIDD corroborating the echocardiographic and histopathological findings. Natriuretic peptides are a family of structurally related peptides involved in the integrated control of renal and cardiovascular function (21). Natriuretic peptides decrease cardiac preload, suppress renin and aldosterone secretion, and exert natriuresis. ANP and BNP are released by myocardial tissue primarily in response to increased stretch of the atrial and myocardial walls (22). Interestingly, canine studies show that proANP is also increased in severe but asymptomatic stages of MR as well as in dogs with clinical signs of congestive heart failure due to MMVD (5). Our study shows that, in agreement with what is seen in both human and canine patients with MV insufficiency (23), proANP can be used as a marker of LV hypertrophy also in this experimental porcine model. Moreover, it underlines that the increase in proANP is strictly correlated with the development of MV insufficiency and the corresponding LV hypertrophy as this model was not influenced by co-morbidities or environmental variations as is the case with patients.

Human and canine heart failure patients (including patients suffering from valvular disease) show endothelial dysfunction and remodeling of vessels (4, 24, 25). Several studies in human patients demonstrate correlations between ADMA, endothelial dysfunction, and cardiovascular risk (6). The clinical role of SDMA (structural isomer of ADMA) remains unclear. SDMA is almost completely eliminated by renal excretion and is a good marker of renal function (26). Furthermore, SDMA is of clinical significance as an independent cardiovascular risk factor associated with increased cardiovascular mortality and N-terminal pro-B-type natriuretic peptide (27). Because pig blood urea nitrogen and creatinine values did not differ significantly between groups, indicating similar renal function, it is likely that the plasma SDMA concentration was affected by increasing MR and more subtle renal changes that are often a consequence of MR and cardiac dysfunction.

Enzymatic imbalances are involved in accumulation of extracellular matrix in affected valves and myocardium. MMPs are Ca^2^^+^- and Zn^2^^+^-dependent proteases expressed in valvular interstitial cells and some isoforms are upregulated in severe canine MMVD (8, 28). MMPs are involved in the regulation of TGFB isoforms (*TGFB1*, *B2*, and *B3*). In the heart, TGFB stimulates genes responsible for controlling fibrosis, angiogenesis, cell proliferation, differentiation, migration, and apoptosis (29). In advanced canine MMVD, activated valvular interstitial cells strongly express *TGFB1* and *TGFB3*, indicating involvement in the myofibroblast-like differentiation of these cells (28, 30). Little is known about the regulation of the myocardial changes associated with MR (4). In this study, we examined several valvular and myocardial key genes (*eNOS*, *INOS*, *MMP9*, *MMP14*, *TGFB1*, *TGFB2*, *TGFB3*, *ANP*, and *BNP*), which are affected in animal models of MMVD. However, the hemodynamic changes inducing MR in this experimental model did not lead to significant de-regulation of the expression of any of these genes. The expression of *ANP* was significantly increased in AP tissue from sMR pigs compared with mMR pigs. However, as the *ANP* expression in AP did not differ significantly between sMR and control pigs, it cannot be concluded whether these changes are related to MR or the plasma ANP concentration. The results of the qPCR analysis support the plasma ADMA measurements indicating that endothelial function and local *eNOS* and *iNOS* expression in the heart were not affected by the experimentally induced MR.

The pigs in this study mainly developed asymptomatic MR over a period of 8 weeks, and although six pigs had MR >50%, they did not require any treatment and did not suffer from congestive heart failure. *MMP* and *TGFB* expressions are affected in canine hearts with severe MMVD (8, 9, 30). These proteins seem to be involved in remodeling and cellular repair processes. It is likely that these genes are not affected until later stages of MR with extensive cardiac remodeling. Future studies including more pigs developing sMR over a longer period of time are needed to clarify this point.

Hereby, we also investigated the expression profile of relevant microRNAs primarily associated with cardiac hypertrophy. Our results show that in the current model, two of the investigated microRNAs are differentially expressed between the three experimental groups of animals. While *miR-21* is upregulated in MV of the sMR group, *miR-133a* is downregulated in MV of the sMR group. *miR-21* is de-regulated in many pathological conditions as cancers or heart diseases. The fact that *miR-21* is more expressed in MV than in LV and AP can be explained by the valvular changes during MR, with the extent of changes depending on the severity of the MR (31). Therefore, pigs with sMR (MR >50%) would experience more pathological changes translating in upregulation of the expression of the *miR-21* in the sMR group. The upregulation of *miR-21* correlated significantly with the increase in plasma proANP concentration, which could indicate that both genes belong to the same network influencing MV remodeling.

*miR-133a* shows differential expression between heart locations and between experimental groups in our study, being downregulated in MV of the sMR group. *miR-133a* is a known microRNA abundant in cardiac and skeletal muscle (32). It has been described to be involved in cell specification, differentiation, and development. This microRNA is downregulated during cardiac hypertrophy (33, 34, 35) in agreement with our results. Its antiapoptotic role in cardiac hypertrophy was confirmed in a transgenic mouse model (36).

The expression results may reflect the consequences of the secondary hypertrophy in the left ventricle and not of the MR itself; therefore, it could be an indirect consequence more than a direct one. In our study, the microRNAs respond faster than the valvular and myocardial key genes, showing more sensitivity to slight changes in the initial stages of MR and one could speculate that microRNAs regulate initial stages of ventricular remodeling in connection with MR. Further studies are needed to characterize their full relevance in the development of MR.

In conclusion, plasma proANP and SDMA levels and cardiac expression of *miR-21* and *miR-133a* are associated with severity of MR in the current experimental pig model. All the affected markers are primarily associated with myocardial changes and are most likely reflecting the secondary LV hypertrophy following MR. This suggests that hemodynamic changes associated with induced MR in this animal model may especially mimic the early myocardial consequences of asymptomatic MR.

## Supplementary data

This is linked to the online version of the paper at http://dx.doi.org/10.1530/EC-13-0051.

## Figures and Tables

**Table 1:** *In vivo* characteristics of the CON, mMR, and sMR experimental pig groups. Data are expressed as median±interquartile range.

	**CON**	**mMR**	**sMR**
*n*	12	10	6
LVIDd (mm)	40.2 (37.1–43.1)	45.4 (38.6–49.1)*	51.0 (46.4–67.0)^†^
Plasma proANP (pmol/l)	656 (528–733)^11^	746 (466–1177)	1145 (1043–1330)^†^^,^^‡^
Plasma ADMA (μmol/l)	1.17 (1.13–1.39)^11^	1.23 (1.14–1.39)	1.28 (1.12–1.42)
Plasma SDMA (μmol/l)	0.87 (0.79–1.02)^11^	0.88 (0.80–0.99)	1.10 (1.00–1.21)^†^^,^^‡^
Plasma l-arginine (μmol/l)	117.6 (106.1–123.5)^11^	123.3 (108.0–134.9)	128.6 (104.1–151.1)
Plasma creatinine (μmol/l)	115.5 (108.8–119.3)^10^	116.5 (102.3–125.3)^8^	114 (110.0–145.0)^5^
Plasma BUN (mmol/l)	3.06 (2.25–3.74)^10^	2.96 (2.63–4.16)^8^	3.49 (3.37–4.55)^5^
Left ventricular weight (g)	88.2 (82.6–104.4)	102.7 (99.7–119.7)	114.9 (102.3–161.9)*

CON, control; mMR, mild mitral regurgitation (10%<MR≤50%); sMR, severe MR (MR >50%); LVIDd, left ventricular end-diastolic diameter; proANP, pro-atrial natriuretic peptide; ADMA, asymmetric dimethylarginine; SDMA, symmetric dimethylarginine; BUN, blood urea nitrogen. Superscript numbers are the number of animals from which the median in question has been calculated in case of missing samples. *P<0.05 compared with control; ^†^*P*<0.005 compared with control groups; and ^‡^*P*<0.05 compared with mMR groups. Data regarding LVIDd and left ventricular weight have been published previously in reference (13).

**Table 2:** Histopathological characteristics of the valvular changes in CON, mMR, and sMR pig groups.

	**CON**	**mMR**	**sMR**
*n*	11	8	5
No lesions	5 (45%)	4 (50%)	0 (0%)
Focal/minor lesions	6 (55%)	1 (12.5%)	1 (20%)
Moderate lesions	0 (0%)	1 (12.5%)	2 (40%)
Extensive lesions	0 (0%)	2 (25%)	2 (40%)

CON, control; mMR, mild mitral regurgitation (10%<MR≤50%); sMR, severe MR (MR >50%).

## References

[1] Guthrie RB, Edwards JE (1976). Pathology of the myxomatous mitral value. Nature, secondary changes and complications. Minnesota Medicine.

[2] Curtin RJ & Griffin BP. Mitral valve disease: stenosis and regurgitation. In *Cleveland Clinic: Current Clinical Medicine*, 2nd edn. Philadelphia, PA, USA: Saunders Elsevier, 2009.

[3] Pedersen HD, Häggström J (2000). Mitral valve prolapse in the dog: a model of mitral valve prolapse in man. Cardiovascular Research.

[4] Falk T, Jönsson L, Olsen LH, Pedersen HD (2006). Arteriosclerotic changes in the myocardium, lung, and kidney in dogs with chronic congestive heart failure and myxomatous mitral valve disease. Cardiovascular Pathology.

[5] Tarnow I, Olsen LH, Kvart C, Hoglund K, Moesgaard SG, Kamstrup TS, Pedersen HD, Häggström J (2009). Predictive value of natriuretic peptides in dogs with mitral valve disease. Veterinary Journal.

[6] Päivä H, Kähönen M, Lehtimäki T, Alfthan G, Viikari J, Laaksonen R, Hutri-Kähönen N, Laitinen T, Taittonen L, Raitakari OT (2010). Levels of asymmetrical dimethylarginine are predictive of brachial artery flow-mediated dilation 6 years later. The Cardiovascular Risk in Young Finns Study. Atherosclerosis.

[7] Buchanan JW (1977). Chronic valvular disease (endocardiosis) in dogs. Advances in Veterinary Science and Comparative Medicine.

[8] Aupperle H, Thielebein J, Kiefer B, Marz I, Dinges G, Schoon HA (2009). An immunohistochemical study of the role of matrix metalloproteinases and their tissue inhibitors in chronic mitral valvular disease (valvular endocardiosis) in dogs. Veterinary Journal.

[9] Aupperle H, Thielebein J, Kiefer B, Marz I, Dinges G, Schoon HA, Schubert A (2009). Expression of genes encoding matrix metalloproteinases (MMPs) and their tissue inhibitors (TIMPs) in normal and diseased canine mitral valves. Journal of Comparative Physiology.

[10] Aupperle H, Disatian S (2012). Pathology, protein expression and signaling in myxomatous mitral valve degeneration: comparison of dogs and humans. Journal of Veterinary Cardiology.

[11] Leroux AA, Moonen ML, Pierard LA, Kolh P, Amory H (2012). Animal models of mitral regurgitation induced by mitral valve chordae tendineae rupture. Journal of Heart Valve Disease.

[12] Bjerre M, Jensen H, Andersen JD, Ringgaard S, Smerup M, Wierup P, Hasenkam JM, Nielsen SL (2008). Chronic ischemic mitral regurgitation induced in pigs by catheter-based coronary artery occlusion. Journal of Heart Valve Disease.

[13] Ravn N, Zois NE, Moesgaard S, Honge JL, Smerup MH, Hasenkam JM, Sloth E, Cremer S & Olsen LH. Rapid development of left ventricular hypertrophy in a chronic non-ischemic mitral regurgitation porcine model. Poster at the The Society for Heart Valve Disease (SHVD) biennial meeting, Barcelona, Spain, 2011.

[14] Hunter I, Rehfeld JF, Goetze JP (2011). Measurement of the total proANP product in mammals by processing independent analysis. Journal of Immunological Methods.

[15] Goetze JP, Hunter I, Lippert SK, Bardram L, Rehfeld JF (2012). Processing-independent analysis of peptide hormones and prohormones in plasma. Frontiers in Bioscience.

[16] Teerlink T, Nijveldt RJ, de Jong S, van Leeuwen PA (2002). Determination of arginine, asymmetric dimethylarginine, and symmetric dimethylarginine in human plasma and other biological samples by high-performance liquid chromatography. Analytical Biochemistry.

[17] de Jong S, Teerlink T (2006). Analysis of asymmetric dimethylarginine in plasma by HPLC using a monolithic column. Analytical Biochemistry.

[18] Balcells I, Cirera S, Busk PK (2011). Specific and sensitive quantitative RT-PCR of miRNAs with DNA primers. BMC Biotechnology.

[19] Moesgaard SG, Olsen LH, Aasted B, Viuff BM, Pedersen LG, Pedersen HD, Harrison AP (2007). Direct measurements of nitric oxide release in relation to expression of endothelial nitric oxide synthase in isolated porcine mitral valves. Journal of Veterinary Medicine. A, Physiology, Pathology, Clinical Medicine.

[20] Nygard AB, Jørgensen CB, Cirera S, Fredholm M (2007). Selection of reference genes for gene – expression studies in pig tissues using SYBR green qPCR. BMC Molecular Biology.

[21] Levin ER, Gardner DG, Samson WK (1998). Mechanisms of disease – natriuretic peptides. New England Journal of Medicine.

[22] Roncon-Albuquerque R, Vasconcelos M, Lourenco AP, Brandao-Nogueira A, Teles A, Henriques-Coelho T, Leite-Moreira AF (2006). Acute changes of biventricular gene expression in volume and right ventricular pressure overload. Life Sciences.

[23] Wolf J, Gerlach N, Weber K, Klima A, Wess G (2012). Lowered N-terminal pro-B-type natriuretic peptide levels in response to treatment predict survival in dogs with symptomatic mitral valve disease. Journal of Veterinary Cardiology.

[24] Nakamura M, Yoshida H, Arakawa N, Mizunuma Y, Makita S, Hiramori K (1996). Endothelium-dependent vasodilatation is not selectively impaired in patients with chronic heart failure secondary to valvular heart disease and congenital heart disease. European Heart Journal.

[25] Moesgaard SG, Klostergaard C, Zois NE, Teerlink T, Molin M, Falk T, Rasmussen CE, Luis Fuentes V, Jones ID, Olsen LH (2012). Flow-mediated vasodilation measurements in Cavalier King Charles Spaniels with increasing severity of myxomatous mitral valve disease. Journal of Veterinary Internal Medicine.

[26] Kielstein JT, Salpeter SR, Bode-Boeger SM, Cooke JP, Fliser D (2006). Symmetric dimethylarginine (SDMA) as endogenous marker of renal function: a meta-analysis. Nephrology, Dialysis, Transplantation.

[27] Meinitzer A, Kielstein JT, Pilz S, Drechsler C, Ritz E, Boehm BO, Winkelmann BR, März W (2011). Symmetrical and asymmetrical dimethylarginine as predictors for mortality in patients referred for coronary angiography: the Ludwigshafen Risk and Cardiovascular Health study. Clinical Chemistry.

[28] Obayashi K, Miyagawa-Tomita S, Matsumoto H, Koyama H, Nakanishi T, Hirose H (2011). Effects of transforming growth factor-β3 and matrix metalloproteinase-3 on the pathogenesis of chronic mitral valvular disease in dogs. American Journal of Veterinary Research.

[29] Dobaczewski M, Chen W, Frangogiannis NG (2011). Transforming growth factor (TGF)-β signaling in cardiac remodelling. Journal of Molecular and Cellular Cardiology.

[30] Aupperle H, März I, Thielebein J, Schoon HA (2008). Expression of transforming growth factor-β1, -β2 and -β3 in normal and diseased canine mitral valves. Journal of Comparative Physiology.

[31] Moustafa SE, Mookadam F, Alharthi M, Kansal M, Bansal RC, Chandrasekaran K (2012). Mitral annular geometry in normal and myxomatous mitral valves: three-dimensional transesophageal echocardiographic quantification. Journal of Heart Valve Disease.

[32] Abdellatif M (2010). The role of microRNA-133 in cardiac hypertrophy uncovered. Circulation Research.

[33] van Rooij E, Sutherland LB, Liu N, Williams AH, McAnally J, Gerard RD, Richardson JA, Olson EN (2006). A signature pattern of stress-responsive microRNAs that can evoke cardiac hypertrophy and heart failure. PNAS.

[34] Carè A, Catalucci D, Felicetti F, Bonci D, Addario A, Gallo P, Bang ML, Segnalini P, Gu Y, Dalton ND (2007). MicroRNA-133 controls cardiac hypertrophy. Nature Medicine.

[35] Cheng Y, Ji R, Yue J, Yang J, Liu X, Chen H, Dean DB, Zhang C (2007). MicroRNAs are aberrantly expressed in hypertrophic heart: do they play a role in cardiac hypertrophy?. American Journal of Pathology.

[36] Matkovich SJ, Wang W, Tu Y, Eschenbacher WH, Dorn LE, Condorelli G, Diwan A, Nerbonne JM, Dorn GW (2010). MicroRNA-133a protects against myocardial fibrosis and modulates electrical repolarization without affecting hypertrophy in pressure-overloaded adult hearts. Circulation Research.

